# Impact of Isolated Exercise-Induced Small Airway Dysfunction on Exercise Performance in Professional Male Cyclists

**DOI:** 10.3390/sports12040112

**Published:** 2024-04-19

**Authors:** Konstantinos M. Pigakis, Vasileios T. Stavrou, Aggeliki K. Kontopodi, Ioannis Pantazopoulos, Zoe Daniil, Konstantinos Gourgoulianis

**Affiliations:** 1 Department of Respiratory & Critical Care Medicine, Creta Interclinic, 71304 Heraklion, Greece; 2 Laboratory of Cardiopulmonary Testing and Pulmonary Rehabilitation, Faculty of Medicine, University of Thessaly, 41110 Larissa, Greece; vasileiosstavrou@hotmail.com (V.T.S.); zdaniil@med.uth.gr (Z.D.); kgourg@med.uth.gr (K.G.); 3 Department of Emergency Medicine, Faculty of Medicine, University of Thessaly, 41110 Larissa, Greece; pantazopoulosioannis@yahoo.com; 4 Department of Respiratory Medicine, Faculty of Medicine, University of Thessaly, 41110 Larissa, Greece

**Keywords:** exercise-induced bronchoconstriction, isolated exercise-induced small airway dysfunction, exercise-induced airway injury, pulmonary function tests, cardiopulmonary exercise testing, spirometry, small airway disease

## Abstract

Background: Professional cycling puts significant demands on the respiratory system. Exercise-induced bronchoconstriction (EIB) is a common problem in professional athletes. Small airways may be affected in isolation or in combination with a reduction in forced expiratory volume at the first second (FEV_1_). This study aimed to investigate isolated exercise-induced small airway dysfunction (SAD) in professional cyclists and assess the impact of this phenomenon on exercise capacity in this population. Materials and Methods: This research was conducted on professional cyclists with no history of asthma or atopy. Anthropometric characteristics were recorded, the training age was determined, and spirometry and specific markers, such as fractional exhaled nitric oxide (FeNO) and immunoglobulin E (IgE), were measured for all participants. All of the cyclists underwent cardiopulmonary exercise testing (CPET) followed by spirometry. Results: Compared with the controls, 1-FEV_3_/FVC (the fraction of the FVC that was not expired during the first 3 s of the FVC) was greater in athletes with EIB, but also in those with isolated exercise-induced SAD. The exercise capacity was lower in cyclists with isolated exercise-induced SAD than in the controls, but was similar to that in cyclists with EIB. This phenomenon appeared to be associated with a worse ventilatory reserve (VE/MVV%). Conclusions: According to our data, it appears that professional cyclists may experience no beneficial impacts on their respiratory system. Strenuous endurance exercise can induce airway injury, which is followed by a restorative process. The repeated cycle of injury and repair can trigger the release of pro-inflammatory mediators, the disruption of the airway epithelial barrier, and plasma exudation, which gradually give rise to airway hyper-responsiveness, exercise-induced bronchoconstriction, intrabronchial inflammation, peribronchial fibrosis, and respiratory symptoms. The small airways may be affected in isolation or in combination with a reduction in FEV_1_. Cyclists with isolated exercise-induced SAD had lower exercise capacity than those in the control group.

## 1. Introduction

Cycling is considered one of the most challenging endurance sports [1,2,3]. Competitive cycling is extremely demanding, and it is known that significant pressure is exerted on the respiratory system during periods of high-intensity exercise [4,5], routinely exposing one to multiple environmental conditions, variations in temperature and humidity, aeroallergens, and particles resulting from the combustion process that takes place in engines. Competitive cycling may lead to airway alterations, which are prevalent in up to one in five elite endurance athletes [6,7,8].

Many professional cyclists frequently experience a feeling of shortness of breath, end-race coughing, and a sense of having “smaller lungs” during or after competition. Some studies support that lung function can worsen following endurance sporting events [3,9]. These studies aimed to examine exercise-induced bronchoconstriction (EIB) in athletes and determine whether changes in FEV_1_ are linked with respiratory symptoms, training volume, race time, and exercise capacity. In a first study [9], participants’ pulmonary function was assessed before a race, 8–10 min after the race (post-test 1), and the day after the race (post-test 2). The results showed that 46% of the participants had EIB at post-test 1 and 28% had EIB at post-test 2. The criterion for EIB diagnosis was a decrease in FEV_1_ of ≥10% after exercise. A second study [3] aimed to examine the impact of systemic hydration on lung function and to determine whether it could reverse dehydration-induced changes in lung function. This research was conducted among professional cyclists without a prior history of asthma or atopy. The lung function of participants was assessed before a cardiopulmonary exercise test (CPET) and at multiple intervals (at the 3rd, 5th, 10th, 15th, and 30th min) following the CPET. According to the criteria for EIB, i.e., a reduction in FEV_1_ of ≥10% after exercise [10], 30% of the participants presented with EIB in post-exercise spirometry.

Exercise-induced bronchoconstriction refers to transient airway contraction following intense exercise, even in individuals with no prior history of asthma [11,12]. This condition is common among elite athletes and may limit their performance [12,13,14,15,16], suggesting that environmental factors play a more important role than genetic factors. Environmental factors may also have a supplementary impact on the underlying genetic tendency toward bronchoconstriction and may be independent and significant etiological factors [12]. EIB is often classified as either EIB with asthma (EIB in asthmatic individuals) or EIB without asthma (EIB activated by exercise in individuals without other symptoms of asthma) [12]. EIB affects 20% to 50% of professional athletes, especially those who engage in high-intensity aerobic activity [12,17,18]. Hyperventilation [19] and a hyperosmotic environment in the airway are the primary triggers for EIB [19,20]. In professional athletes, hyperventilation can result in airway epithelial damage and the release of special mediators that activate bronchoconstriction [11,21,22,23]. Some researchers have demonstrated that training in endurance sports may not only boost bronchoconstriction, but can also lead to airway hyper-reactivity outside of training and exercise conditions or to persistent airway remodeling [24,25,26].

The epithelium of small airways appears to be the most vulnerable to damage and repair, as observed in mouse studies [16]. Exercise can affect the small airways alone, without the classical diagnosis of EIB, that is, a reduction of ≥10% in FEV_1_ after exercise [3]. The impact of isolated SAD on exercise performance among athletes without EIB is not yet fully understood, as it is difficult to comprehend due to the rarity of subjects presenting with isolated small airway dysfunction. This has caused some to refer to the small airways as the ‘quiet zone’ [27]. The small airways are peripheral airways that are localized from the seventh or eighth generation of airways to the bronchioles, with an inside diameter of less than 2 mm. Their walls mostly lack cartilage, their entire cross-sectional area is large, and their airflow velocity is slow. As such, small airways are more vulnerable to injury due to toxic particles, which can cause obstruction [28]. Studies investigating the relationship between small airway dysfunction and exercise capacity have focused on older individuals with lung diseases and tobacco or environmental exposure [29,30], where relationships are confused by the existence of gas exchange and large airway defects and are overstated by the loss of elastic recoil inherent to aging [29,30].

There are various techniques for detecting SAD, yet there is no agreement on the gold standard for diagnosis. Spirometry is the most commonly used method for evaluating pulmonary ventilation. The indicators of forced expiratory flow are the most commonly used to estimate the function of small airways. Expiratory flow-volume loops (EFVLs) help detect asthma [31,32]. Imaging studies have shown robust correlations between EFVL-derived spirometric indices and some measures of airway obstruction [33]. An extensive review of EFVL-derived spirometric indices revealed the severity of obstruction and the size of the airways affected [34]. Forced expiratory flows evaluated at the mid-portion of the expiratory flow-volume loop, namely, FEF_50_%, FEF_75_%, and FEF_25–75_%, were effective in measuring airway obstruction and airflow limitation [35]. It is accepted that SAD is present when two of the above three parameters are lower than 65% of the predicted values [36]. However, the American Thoracic Society does not suggest the application of these indicators for the determination of SAD [36]. The FEF_25–75_ may be used to detect individuals with early COPD who have clinical symptoms, but whose spirometry has not yet detected the obstruction, recommending that it may be used as a marker of SAD [37]. Additionally, there are various other markers of small airway function with better punctuality. The forced expiratory volume in the third second (FEV_3_), the FEV_3_/FVC ratio, and the 1-FEV_3_/FVC are correlated with air trapping and hyperinflation [38]. The 1-FEV_3_/FVC is estimated to demonstrate the residual un-exhaled vital capacity at the end of the third second [1 − FEV_3_/FVC = (FVC − FEV_3_)/FVC].

Plethysmography is a technique used to measure lung volume, and it cannot be measured via spirometry. Air trapping and hyperinflation assessment are often performed using the evaluation of the residual volume (RV) and RV/TLC (total lung capacity) ratio [39]. Impulse oscillometry (IOS) is a noninvasive method for assessing lung function, and it can be used to assess small airways. High-resolution computed tomography (HRCT) is a technique that offers anatomical information for the airways. However, its use has been restricted due to radiation exposure. Magnetic resonance imaging (MRI) is rarely employed for the respiratory system due to technical restrictions, but hyperpolarized MRI is an area of increasing interest. It has no radiation risk, but it is an expensive method and has only been conducted in research centers.

To date, we have not found any studies examining the occurrence of isolated exercise-induced SAD in athletes who have normal pre-exercise spirometry results and do not meet the post-exercise spirometry criteria for EIB. This study is based on data from earlier research [3]. The study design used in the previous research work is still applicable to this investigation, which has different research questions and results that were not the focus of the first study. The data were analyzed differently in the two studies, and the conclusions are also distinct. The protocol of the previous study divided the methodology into two phases: before and after the hydration of the athletes. The findings of the previous study support that systemic hydration has a positive effect on both VO_2 max_ and pulmonary function in professional cyclists, and it is effective in preventing reductions in their pulmonary function after exercise. In the present study, we used the data obtained from the first phase (before hydration). This research aimed to investigate isolated exercise-induced small airway dysfunction in professional cyclists and assess the impact of this phenomenon on exercise performance in this population.

## 2. Materials and Methods

This study is based on data from earlier research [3], which were analyzed differently. The protocol of the previous study divided the methodology into two phases: before and after hydration of the athletes. In the present study, we used the data obtained from the first phase (before hydration).

A total of one hundred professional male cyclists (100) were called to participate in this research. All of the participants were used to cycling for more than 15 h every week, with sessions lasting at least 3 to 4 h. They were required to refrain from exercise for 48 h before attending a session and to arrive at the laboratory between 9:00 a.m. and 2:00 p.m. The laboratory had an air purification system (Health-Way Deluxe, DFS Technology-VOC Filter, New York, NY, USA) to ensure that the air was clean and safe. The study protocol was approved by the ethics committee of Creta InterClinic Hospital (registration number: 129/16-09-2020), and all subjects provided consent in accordance with the Helsinki Declaration and the regulations of the European Parliament and Council of the European Union for the collection and use of their data.

This research was carried out from October 2020 to January 2021 and involved members of two professional cycling teams in Crete. The inclusion criteria for the study were as follows: age between 18 and 35 years, absence of a history of asthma as confirmed by medical records and spirometry, and a training age of more than 3 years in the sport. The exclusion criteria included female gender, history of smoking, craniofacial and upper airway abnormalities (with a possibility of incorrect application of the ergospirometry mask), injuries sustained within the last 12 months, anemia (Hb < 13.5 g/dL), seasonal allergies affecting the upper and lower respiratory systems, high levels of FeNO (>25 ppb), and high levels of IgE (>100 UI/mL).

Notably, females were excluded from the research due to physiological differences between the sexes, such as differences in reproductive endocrinology, the menstrual cycle, and hormonal contraceptive use. These factors may affect exercise capacity and disturb the objective accuracy of CPET results.

A detailed medical history was taken from all the participants. All cyclists were free from respiratory infection for at least two weeks before entry into the study. Additionally, a clinical examination that included recording demographic and anthropometric parameters, such as height, body mass, and body mass index (BMI), and the determination of the participants’ training age was conducted. All subjects were tested using spirometry, and specific markers, such as FeNO and IgE, were measured. Finally, the participants underwent a cardiopulmonary exercise test (CPET), followed by spirometry at the 3rd, 5th, 10th, 15th, and 30th minutes after the completion of the CPET.

Body mass (kg) and height (cm) were evaluated following the manual reference for anthropometric standardization [40]. All participants wore light clothes and were barefoot (Table 1).

For the needs of the present study, lung function was assessed via spirometry and flow-volume loop analysis. The study protocol required us to evaluate lung function at regular intervals after CPET (at the 3rd, 5th, 10th, 15th, and 30th minutes). Thus, there was limited time to employ additional diagnostic tests other than spirometry. Pulmonary function was evaluated following the ATS/ERS guidelines [36] on a spirometer (Ergocard Clinical with ExpAir E01-10, Medisoft Group, Namur, Belgium). We recorded the following values: forced vital capacity (FVC), FEV_1_, FEV_3_ (forced expiratory volume in 3 s), FEF_25–75_ (forced mid-expiratory flow), FEF_50_, FEF_75_, and the FEV_1_/FVC and FEV_3_/FVC ratios.

The cardiopulmonary exercise test was conducted using a cycloergometer (Ergocardr clinical with ExpAir Software, Medisoft Group, Namur, Belgium). The study protocol involved a two-minute rest period, followed by one minute of warm-up pedaling against a minimal load of 20 watts, which progressively increased by 50 watts every three minutes. The indoor circumstances during the CPET were constant, with a temperature of 24–26 °C and a humidity of 45–50%. Athletes were required to wear appropriate sports clothing for cycling, and they were free to have their regular breakfast three hours prior to the test. They were also asked to avoid exercising 48 h before the test. The test duration was until exhaustion, with continuous monitoring of the heart rate, electrocardiogram, oxygen saturation, and blood pressure being recorded every three minutes. The indications for early test termination included myocardial ischemia, complex ventricular premature beats, grade 2 or 3 atrioventricular block, a sudden decrease in blood pressure by more than 20 mmHg, or increased blood pressure (>220/120 mmHg) [12,41]. During the test, oxygen consumption (VO_2_), carbon dioxide production (VCO_2_), tidal volume (VT), respiratory rate (RR), and minute ventilation (VE) were directly measured.

The statistical analysis was conducted using IBM SPSS Statistics 26.0 (SPSS Inc., version 26, Chicago, IL, USA). A *p*-value of less than 0.05 was assumed to indicate statistical significance. Categorical variables are expressed as counts and percentages. For continuous variables, data normality was evaluated using the Kolmogorov–Smirnov test. Relationships between continuous variables were measured using Pearson’s correlation coefficients. A paired-sample *t*-test was used to estimate the differences in spirometry before and after the CPET. An independent-sample *t*-test was applied to compare the means of two independent samples. One-way analysis of variance or the Kruskal–Wallis test was used to evaluate differences among the control, isolated exercise-induced SAD, and EIB groups adjusted for age, training age, and BMI to assess the robustness of the associations.

## 3. Results

**Figure 1:**
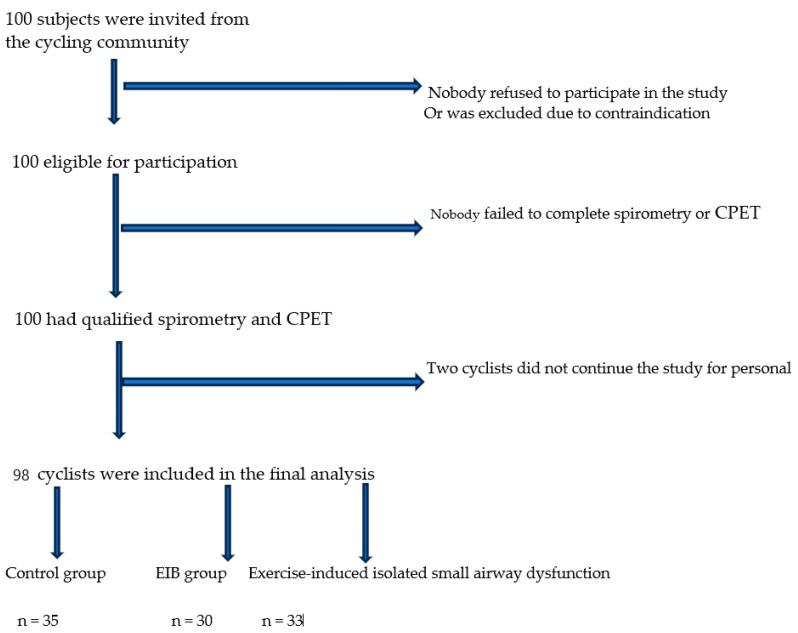
Flowchart illustrating the athlete selection procedure. CPET, cardiopulmonary exercise test; EIB, exercise-induced bronchoconstriction.

One hundred participants (n = 100) were recruited from the professional cycling community. All participants had acceptable spirometry and CPET results and were included in the final assessment. The control group was defined as having normal spirometry after the CPET (ΔFEV_1_ < 10% after the exercise test), the EIB group was defined as having a percentage drop in FEV_1_ (ΔFEV_1_) of ≥10% after the exercise test, and the isolated exercise-induced small airway dysfunction group was defined as having a percentage drop in FEF_25–75_ (ΔFEF_25–75_) of ≥25% after the exercise test (Figure 1).

All subjects were young cyclists with a mean age of 27.0 ± 5.0 years, while the median age was 30.0 years. The BMI of the athletes ranged from 21.5 to 26.8 kg/m^2^ and the mean BMI was 23.8 ± 1.4 kg/m^2^. The baseline demographics and laboratory results obtained during deployment are listed in Table 1.

The results of baseline spirometry are listed in Table 2. There were no abnormalities in the baseline test. In addition, Table 2 presents the demographic characteristics and spirometric results of the participants by study group after the CPET. The control group was defined as having normal spirometry after the CPET, the EIB group was defined as those meeting the EIB criteria with or without small airway dysfunction, and the isolated exercise-induced small airway dysfunction group was defined as those with isolated small airway dysfunction. The baseline characteristics of the participants before the CPET were compared with the results of the participants in each study group after the CPET. Upon separately comparing each group with the control group, the following data were observed. All post-exercise spirometric values, except for the FVC, FEV_1_, and FEV_1_/FVC, were significantly lower in the isolated exercise-induced SAD group than in the controls. In the EIB group, all values, except for the FVC, were significantly lower than those in the controls. There were significant differences in the FEV_1_, FEV_3_, FEV_1_/FVC, 1-FEV_3_/FVC, and MVV between the exercise-induced ISAD and EIB groups.

Table 3 presents the CPET results among the three groups of participants. The VO_2_ max was significantly lower in the isolated exercise-induced SAD and EIB groups than in the controls. The VO_2_ max was lower in the EIB group than in the exercise-induced ISAD group, but the difference was not significant. The anaerobic threshold (VO_2_ AT%) was also lower in the isolated exercise-induced SAD and EIB groups than in the controls, but, again, there was no significant difference between the two groups. There were no significant differences in the O_2_-pulse (VO_2_/HR) between the groups. The minute ventilation (VE) was significantly greater in both the SAD and EIB groups than in the controls, with the EIB group having a slightly greater VE than that of the SAD group. The ventilatory equivalent for carbon dioxide (VE/VCO_2_ ratio) was greater in the EIB group than in the control group. The difference was statistically significant. However, the VE/VCO_2_ ratio was also greater in the SAD group than in the control group, but the difference was not significant. Between the SAD and EIB groups, the VE/VCO_2_ ratio was significantly greater in the EIB group. The ventilatory reserve (VE/MVV%) was significantly greater in both the SAD and EIB groups than in the controls. However, the VE/MVV% was significantly greater in the EIB group than in the SAD group. The respiratory exchange ratio (RER) was significantly lower in the SAD and EIB groups than in the controls. Moreover, the RER was significantly lower in the EIB group than in the SAD group, but the difference was not significant (Table 3).

Using the controls as the reference group, the VO_2 max_ was compared between the groups. The data were adjusted for age, BMI, and training age. Multiple linear regression analysis revealed that the VO_2_ peak was lower in the ISAD and the EIB groups (Table 4).

## 4. Discussion

This study has two novel findings. Firstly, participants without EIB (who did not meet the spirometric criteria for EIB) but with isolated exercise-induced small airway dysfunction presented lower exercise capacity than those in the controls. However, their exercise performance was similar to that of the EIB group. Secondly, lower exercise capacity in the group with isolated small airway dysfunction appeared to be associated with a poorer ventilatory reserve (VE/MVV%) compared with that of the control group. 

Post-exercise spirometry revealed that cyclists in the ΕΙΒ group had significantly lower FEV_1_ values than those of cyclists in the control and SAD groups. Although their FEV_1_ values were within the normal range, cyclists with ISAD had poorer exercise capacity and breathing reserves during exercise than those of the controls.

Professional athletes train every day to stay in shape and build endurance. However, they may experience a decline in exercise performance despite their hard work due to overtraining, which occurs when they exercise too much without enough recovery time between workouts. This can lead to overtraining syndrome or burnout, where athletes experience a plateau or decline in athletic performance. Continuing to work while experiencing overtraining syndrome can cause more damage to the body and lengthen the necessary recovery time. Recovery from overtraining requires rest and limiting or even stopping training for a determined period. Prevention of overtraining syndrome should be considered by all who interact with endurance athletes. Unfortunately, there is no definitive biomarker available to differentiate functional overreaching from nonfunctional overreaching or nonfunctional overreaching from overtraining. Healthcare providers must still rule out other medical conditions. The differential diagnosis of underperformance, among other things, should also include a thorough examination of the respiratory system to rule out conditions that lead to exercise underperformance. EIB is a serious condition in professional endurance athletes. The symptoms of EIB in this population are nonspecific, and the diagnosis must be established with specific tests [12]. The differential diagnosis of EIB is wide. Many professional athletes with EIB have no history of asthma, indicating that in these cases, environmental agents are more significant than genetic factors. Many investigators have suggested defining EIB with asthma as the manifestation of bronchoconstriction after exercise in asthmatic athletes and EIB without asthma as the manifestation of bronchoconstriction caused by physical activity in individuals without other symptomatology of asthma [12]. Exercise-induced laryngeal dysfunction (EILD) can often present with respiratory symptoms. EILD can appear in isolation or can co-occur with EIB [12]. Gastroesophageal reflux disease and laryngopharyngeal reflux can also be exacerbated by physical activity and imitate respiratory symptoms [12]. Our study is the first in the literature to address small airway dysfunction caused by functional overtraining and how this dysfunction can negatively impact exercise capacity and athletic performance. The small airways seem to be impacted in isolation or in association with a reduction in FEV_1_. Individuals with isolated exercise-induced SAD have a lower exercise capacity. Accurate recognition and early identification of SAD are important because treatment may be able to reverse airway remodeling in athletes and improve their exercise capacity. Clinicians should consider, among other things, the possibility that poor physical condition in endurance athletes may be due to a pathology of the peripheral airways to prevent a misdiagnosis of overtraining, which could have negative consequences on the athlete’s career due to the necessity of abstention from the sport for a long time, leading to a loss of training benefits.

In this research, we demonstrated that the fraction of FVC that does not expire at the end of the first three seconds of the FVC (1-FEV_3_/FVC) was significantly greater in both EIB athletes and those with ISAD than in the controls.

The earliest alteration in airflow limitation is a decrease in the final fragment of the spirogram, while the initial fragment is weakly affected [42]. Later fractions of forced exhalation, i.e., those after FEV_1_, such as FEV_3_, were suggested to define declines in terminal expiratory flow [43,44]. The FEV_3_ and FEV_3_/FVC have been used in recent years [45,46,47]. Later, the concept of the 1-FEV_3_/FVC was introduced as an assessment of the late expiratory fraction in smokers and never-smokers [48]. Our research suggests that this parameter can be employed to evaluate the occurrence of hyperinflation and air trapping in cases where lung volumes cannot be estimated. This was demonstrated in our study, where measurements had to be taken in a very short time after one another.

According to one study, the 1-FEV_3_/FVC is a better measure of airflow limitation than the FEF_25–75_, as false-negative and false-positive test results might often occur when using the FEF_25–75_ [44]. However, in our study, we found that the FEF_25–75_ had a strong correlation with the 1-FEV_3_/FVC ratio in both the overall population of the research and in the subgroups. A previous study revealed that the FEF_25–75_ is more strongly correlated with RV/TLC than with the 1-FEV_3_/FVC, while the 1-FEV_3_/FVC is more strongly correlated with the RV than with the FEF_25–75_ [38].

There are no existing studies estimating the impact of ISAD on exercise performance, especially in athletes. Our investigation is the first attempt to research this theme. Previous studies have demonstrated that exercise capacity is lower in smokers who do not have obstructive spirometry than in nonsmokers, and SAD is one of the causes of this finding [49,50]. Our study on cyclists established this finding and showed that athletes with ISAD are at a greater risk for lower exercise performance. Another study on miners revealed that numerous pathological processes in the small airway can be impacted by dust exposure, and this is assumed to be the primary cause of exercise intolerance [29]. Our research expands these results to elite cyclists and shows that athletes without EIB who have ISAD also have lower exercise capacity, regardless of BMI, age, and training age. This finding has implications for the better identification of the respiratory health of athletes and supports the idea that professional endurance athletes may have a nonbeneficial impact on their small airway function. Individuals with small airway dysfunction may experience air trapping during exercise [51,52], which can negatively affect ventilatory reserves and exercise capacity in athletes. Our research confirms this speculation. We found that the ventilatory reserve was lower in athletes with ISAD than in the controls. We also found that the VO_2_ peak was negatively correlated with the ventilatory reserve in the CPET and with small airway dysfunction in post-exercise spirometry. Consequently, we consider that the lower exercise performance in athletes with ISAD may be related to air trapping during exercise. Our research confirms this speculation. A larger sample size would be needed for the correct estimation of small airway dysfunction in athletes. These ideas require additional study.

We are confident that the lower VO_2_ peak in the isolated exercise-induced small airway dysfunction group was not the result of decreased motivational trying because the athletes continued to exercise until exhaustion. It is widely recognized that age, BMI [53], and, possibly, training age can affect test performance. Therefore, we corrected for confounding factors after performing a univariable analysis to support our conclusions.

This research has some limitations that should be considered. First, the diagnosis of exercise-induced small airway dysfunction was only based on post-exercise spirometry, which is known to be more variable than other methods. Although the FEF_25–75_%, FEF_50_%, and FEF_75_% are commonly employed to assess SAD, they have some limitations. Other than spirometry, there are several techniques for assessing the function of small airways, including impulse oscillometry, high-resolution computed tomography (HRCT), body plethysmography, and gas washout. Additionally, we must acknowledge that the study included only male participants. This was because the menstrual cycle and fluctuations in female sex hormones can affect athletic performance. Therefore, females were excluded from the study to reduce the variability in the results.

## 5. Conclusions

The findings of this study indicate that professional cyclists may experience non-beneficial effects on their respiratory system. Strenuous endurance exercise can induce airway injury, which is followed by a restorative process. The repeated cycle of injury and repair can trigger the release of pro-inflammatory mediators, the disruption of the airway epithelial barrier, and plasma exudation, which gradually give rise to airway hyper-responsiveness, exercise-induced bronchoconstriction, intrabronchial inflammation, peribronchial fibrosis, airway remodeling, and respiratory symptoms. The small airways may be affected in isolation or in combination with a reduction in FEV_1_. Cyclists with isolated exercise-induced SAD had lower exercise capacity than those in the controls. The small airways seem to be impacted independently or in combination with the reduction in FEV_1_. Individuals with isolated exercise-induced SAD have lower exercise capacity regardless of age, training age, and BMI. The role of small airways in EIB still must be elucidated, and more investigations are required to evaluate whether SAD may be considered a kind of EIB in nonasthmatic and nonatopic professional cyclists. An accurate and early diagnosis of SAD is important because treatment reverses airway remodeling in athletes and improves their exercise capacity. A promising marker of air trapping and hyperinflation is the 1-FEV_3_/FVC, which can conveniently be determined from spirometric volumes. We recommend using the 1-FEV_3_/FVC, along with the FEF_25–75_, FEF_50_, and FEF_75_, during spirometry to estimate isolated exercise-induced SAD in athletes. However, future studies involving comparisons among athletes from different endurance sports are warranted to confirm our findings.

## Figures and Tables

**Table 1:** Demographic, somatometric, and laboratory characteristics of the athletes.

Age, years	27.0 ± 5.0
Training age, years	12.0 ± 5.0
Body mass index, kg/m^2^	23.8 ± 1.4
Hemoglobin, g/dL	14.8 ± 1.1
FeNO, ppb	11.0 ± 3.0
IgE, UI/mL	53.0 ± 7.0

**Table 2:** Comparison of the demographic, somatometric, and spirometric characteristics of athletes in each study group after the CPET. The data are presented as the mean ± SD. * *p* < 0.05 vs. the controls. ^†^ *p* < 0.05, EIB group vs. the exercise-induced isolated SAD group.

	Baseline Characteristics (before the CPET) n = 100	Control Group (after the CPET) n = 35	Isolated Exercise-Induced SAD Group (after the CPET) n = 33	EIB Group (after the CPET) n = 30
FVC, L	6.40 ± 0.6	6.10 ± 0.7	6.10 ± 0.70	6.0 ± 0.72
FVC, %	117.1 ± 6.7	112.4 ± 8.9	112.0 ± 4.4	110.0 ± 4.6
FEV_1_, L	5.20 ± 0.4	4.90 ± 0.5	4.73 ± 0.47	4.28 ± 0.47 *^†^
FEV_1_, %	120.0 ± 6.9	118.0 ± 12	117.0 ± 14	109.0 ± 10 *^†^
FEV_3_, L	5.85 ± 0.5	5.65 ± 0.55	5.32 ± 0.4 *	5.10 ± 0.6 *^†^
FEV_3_, %	120.0 ± 6.5	119.0 ± 11.5	115.0 ± 8.5 *	110.0 ± 9.2 *^†^
FEV_1_/FVC	0.81 ± 0.1	0.80 ± 0.7	0.77 ± 0.65	0.71 ± 0.45 *^†^
FEV_3_/FVC	0.91 ± 0.2	0.93 ± 0.8	0.87 ± 0.3 *	0.85 ± 0.6 *
1-FEV_3_/FVC, %	8.6 ± 2.3	7.4 ± 2.5	12.8 ± 3.2 *	15.0 ± 2.8 *^†^
MVV, L	182.0 ± 34.0	171.5 ± 18.9	165.5 ± 16.4 *	149.8 ± 15.2 *^†^
FEF_25–75_, L/s	5.0 ± 1.1	4.40 ± 1.2	1.96 ± 1.4 *	1.76 ± 1.35 *
FEF_25–75_, %	103.1 ± 8.3	90.8 ± 11.5	65.0 ± 10 *	63.0 ± 9.0 *
FEF_50_, L/s	6.20 ± 0.7	7.60 ± 0.7	3.51 ± 0.4 *	3.49 ± 0.3 *
FEF_50_, %	112.0 ± 4.5	138.0 ± 5.3	63.0 ± 3.5 *	63.0 ± 6.0 *
FEF_75_, L/s	3.30 ± 0.4	3.47 ± 0.7	1.62 ± 0.4 *	1.58 ± 0.4 *
FEF_75_, %	127.0 ± 8.0	134.0 ± 7.0	63.0 ± 4.0 *	61.0 ± 7.0 *
Age, years	27.0 ± 5.0	26.2 ± 5.1	29.2 ± 6.1 *	32.0 ± 4.3 *
BMI, kg/m^2^	23.8 ± 1.4	21.9 ± 3.2	22.3 ± 3.1	23.2 ± 3.4

**Table 3:** CPET results among the three groups of participants. * *p* < 0.05 vs. the controls. ^†^ *p* < 0.05, EIB group vs. the exercise-induced isolated SAD group.

	Overall	Controls	SAD	EIB
VO_2 max_, mL/kg/min	65.0 ± 4.4	69.0 ± 2.0	62.0 ± 2.3 *	61.0 ± 2.35 *
Respiratory exchange ratio (RER)	1.20 ± 0.1	1.22 ± 0.1	1.18 ± 0.09 *	1.15 ± 0.11 *
VO_2_ AT% predicted VO_2 max_, %	72.4 ± 5.2	75.5 ± 6.8	72.0 ± 2.8	70.0 ± 6.15
O_2_-pulse (VO_2_/HR), mL/beats/min	19.2 ± 2.5	19.5 ± 2.7	19.2 ± 2.2	18.9 ± 2.5
VE, L/min	120.0 ± 26.0	113.0 ± 28	122.0 ± 27 *	125.0 ± 27.5 *
VE/VCO_2_ ratio (ventilatory efficiency)	27.5 ± 1.3	26.5 ± 1.0	27.0 ± 1.5	29.0 ± 1.5 *^†^
Respiratory reserve (VE/MVV, %)	64.0 ± 1.3	62.0 ± 1.0	74.0 ± 2.0 *	83.0 ± 1.0 *^†^

**Table 4:** Multivariate linear analysis of exercise capacity across the groups.

Variables	Control Group	ISAD Group		EIB Group	
		Β (95% CI)	*p*	Β (95% CI)	*p*
VO_2_ peak	Ref.	−2.4 (−6.2 to −1.4)	0.025	−4.0 (−7.7 to −0.2)	0.039

## Data Availability

The data presented in this study are available on request from the corresponding author. The data are not publicly available due to privacy and ethical restrictions.
